# The Crossroads of Glycoscience, Infection, and Immunology

**DOI:** 10.3389/fmicb.2021.731008

**Published:** 2021-09-27

**Authors:** Tanya R. McKitrick, Margaret E. Ackerman, Robert M. Anthony, Clay S. Bennett, Michael Demetriou, Gregory A. Hudalla, Katharina Ribbeck, Stefan Ruhl, Christina M. Woo, Loretta Yang, Seth J. Zost, Ronald L. Schnaar, Tamara L. Doering

**Affiliations:** ^1^National Center for Functional Glycomics, Harvard Medical School, Boston, MA, United States; ^2^Thayer School of Engineering, Dartmouth College, Hanover, NH, United States; ^3^Center for Immunology and Inflammatory Diseases, Division of Rheumatology, Allergy, and Immunology, Massachusetts General Hospital, Harvard Medical School, Boston, MA, United States; ^4^Department of Chemistry, Tufts University, Medford, MA, United States; ^5^Department of Neurology, Microbiology, and Molecular Genetics, University of California, Irvine, Irvine, CA, United States; ^6^J Crayton Pruitt Family Department of Biomedical Engineering, University of Florida, Gainesville, FL, United States; ^7^Department of Biological Engineering, Massachusetts Institute of Technology, Cambridge, MA, United States; ^8^Department of Oral Biology, University at Buffalo School of Dental Medicine, Buffalo, NY, United States; ^9^Department of Chemistry and Chemical Biology, Harvard University, Cambridge, MA, United States; ^10^Lectenz Bio, Athens, GA, United States; ^11^Vanderbilt Vaccine Center, Vanderbilt University Medical Center, Nashville, TN, United States; ^12^Department of Pharmacology, Johns Hopkins University, Baltimore, MD, United States; ^13^Department of Molecular Microbiology, Washington University School of Medicine, St. Louis, MO, United States

**Keywords:** glycobiology, glycomedicine, glycoscience, host response, infection, immunity, microbial glycans

## Abstract

Advances in experimental capabilities in the glycosciences offer expanding opportunities for discovery in the broad areas of immunology and microbiology. These two disciplines overlap when microbial infection stimulates host immune responses and glycan structures are central in the processes that occur during all such encounters. Microbial glycans mediate host-pathogen interactions by acting as surface receptors or ligands, functioning as virulence factors, impeding host immune responses, or playing other roles in the struggle between host and microbe. In the context of the host, glycosylation drives cell–cell interactions that initiate and regulate the host response and modulates the effects of antibodies and soluble immune mediators. This perspective reports on a workshop organized jointly by the National Institute of Allergy and Infectious Diseases and the National Institute of Dental and Craniofacial Research in May 2020. The conference addressed the use of emerging glycoscience tools and resources to advance investigation of glycans and their roles in microbe-host interactions, immune-mediated diseases, and immune cell recognition and function. Future discoveries in these areas will increase fundamental scientific understanding and have the potential to improve diagnosis and treatment of infections and immune dysregulation.

## Introduction

**Figure 1:**
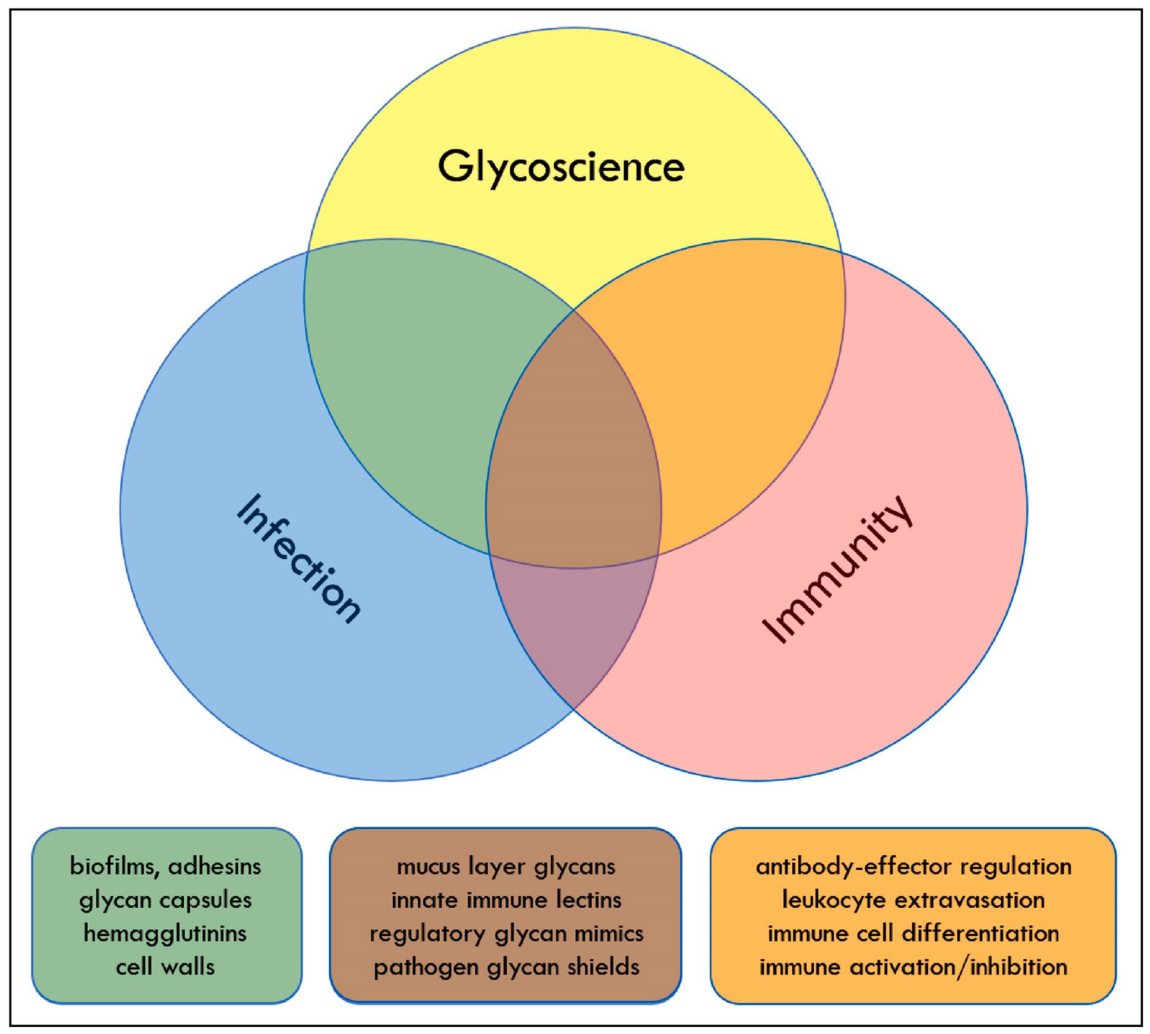
Glycoscience, infection, and immunity overlap in multiple areas that drive pathogen and host function. Color-coded overlap topics mentioned in the text are listed as examples.

During the early months of the COVID-19 pandemic (May 27–28, 2020), an NIH workshop on “Glycoscience and Immunology at the Crossroads of Biology” was convened on-line. The component topics of the workshop were infection, immunity, and glycobiology. Each of these broad areas is the subject of intense scientific investigation, and resulting discoveries have advanced human health. Many studies also occur at the intersections of these fields. For example, infection and immunity represent two views of the events that occur during and after encounters between pathogenic microbes and their hosts. Understanding how these events unfold from each vantage point has been critical for the development of modern immunology and microbiology and for the development of strategies to treat immune dysregulation and infectious disease. This perspective, like the NIH workshop on which it is based, focuses on the overlap of glycoscience with each of these two fields (Figure 1).

Glycans play key roles in infection and immunity: Pathogen glycans may mediate host interactions and stimulate or inhibit host immune responses, while host glycans may serve as specific targets of microbial adhesion molecules or toxins (Varki and Gagneux, 2015). Glycans also act in mediating the host response to infection and in regulating immunity at multiple levels. In all of these roles, their primary function is molecular recognition, as opposed to their structural and dietary cousins (more often termed sugars, saccharides, or carbohydrates).

This brief perspective will use the topics discussed at the NIH workshop as examples to focus attention on emerging areas of research and opportunities in the many areas of infection and immunity where glycans play key roles. It will also highlight the importance of glycoscience tools for scientific progress on these topics and identify areas where investment in basic research efforts will advance knowledge and practice in glycobiology and glycomedicine.

## Glycans in Host-Pathogen Recognition and Disease

Microbial glycans are incredibly diverse and play critical roles in the interactions between infectious agents and their hosts and in the pathogenesis of resulting infections. These compounds frequently constitute much of the microbial cell surface and therefore mediate the initial encounters between pathogen and host cells. Bacteria, for example, are protected by a peptidoglycan cell wall and often display polysaccharide capsules as well as other glycan-containing moieties. The cell walls of fungi are primarily composed of glycan polymers and highly glycosylated proteins. Many parasites display surface coats that are both anchored by glycolipids and abundantly glycosylated. Study of these glycans has revealed novel biological pathways, elucidated pathogenic processes, and led to the development of vaccines and therapeutics.

Microbial glycan structures contribute to pathogenesis by an array of distinct mechanisms. They may physically protect the invading pathogen, mediate cell adherence or protein interactions, transmit signaling information, serve as decoys, or alter the environment to the benefit of the invader, as when biofilm production reduces the efficacy of antibiotics or efficiency of host clearance. Tamara Doering presented the opportunistic eukaryote *Cryptococcus neoformans* as an example of a pathogen whose glycans are critical for the development of disease (Loza and Doering, 2021). This yeast, which is responsible for roughly 200,000 deaths from meningitis each year, elaborates an extracellular capsule that is composed of large (up to millions of daltons) polysaccharides and can comprise >75% of the pathogen volume. The capsule, made primarily of mannose or galactose chains with appendant glucuronic acid and xylose residues, is required for infection and inhibits host cell phagocytosis (Gaylord et al., 2020). Shed capsule components also perturb host immune responses; this material is also the basis for rapid tests that are valuable for diagnosis of this frequently lethal infection.

In addition to glycans produced by microbes themselves, host glycoconjugates are critical in determining the outcomes of host-pathogen interactions. Influenza virus is a compelling example of this dual association of glycobiology and pathogenesis. This virus exploits host glycans by using sialic acid bearing proteins for cell entry (mediated by hemagglutinin) and a sialidase (neuraminidase) to trigger release of budding virions (Gamblin and Skehel, 2010); as a result, species-specific differences in sialic acid isomers impact the host selectivity of various strains. For example, pathogenic human influenza strains all bear hemagglutinins that bind sialic acid linked to the 6-carbon hydroxyl of galactose whereas bird influenza binds to sialic acid when linked to the 3-carbon hydroxyl of galactose. The molecular switch in human to bird specificity can occur when as few as two amino acids in the sialic acid binding site of influenza hemagglutinin are appropriately mutated.

On the flip side, influenza also illustrates how microbial protein glycosylation can impact host defenses. Seth Zost discussed how antigenic drift in the influenza virus hemagglutinin protein may alter its glycosylation, which in turn can change characteristics of the infection, such as infectivity and viral fitness, as well as the efficacy of host antibody responses that neutralize the virus (Zost et al., 2017; Altman et al., 2019). Vaccine efficacy may also change in this scenario, both because the new antigen will induce a distinct antibody response and because protection conferred by prior immunization may be less robust.

The exploitation of host glycans by microbial invaders to advance infection and disease occurs frequently across domains of microbiology. For instance, Stefan Ruhl discussed the contributions of host glycan recognition to the physiology of the oral microbiome. The interactions between lectin-like adhesins on bacteria and complementary glycan motifs on glycoproteins adsorbed to tooth enamel play central roles in initial bacterial colonization. Lectin-glycan binding also facilitates bacterial coadhesion that leads to the formation of microbial biofilms. Glycan-driven bacterial-host interactions are key both to establishing the commensal oral microbiota and to oral disease progression (Thamadilok et al., 2016; Cross and Ruhl, 2018). Host glycans can also significantly impact pathogen behavior by modulating the immediate pathogen environment. As a striking example, Katharina Ribbeck presented the effects on epithelial microbes of host mucus, which is often excluded from experiments performed *in vitro* despite its known role in defense against infection. Her group has shown that mucin-associated glycans influence multiple microbial functions that are central to pathogenic processes of yeast and bacteria, including surface attachment, quorum sensing, virulence gene expression, and biofilm formation. Released O-linked glycans from highly glycosylated mucins, such as MUC5B, retain many of these effects.

## Glycans in Tuning and Control of Immune Responses

As major molecular determinants on cell surfaces, on secreted proteins, and in the extracellular matrix, glycans are well suited to regulate molecular recognition and molecular signaling events. Nowhere is this more evident than in the immune system, where different types of immune cells respond to secreted factors, each other, and molecules in their extracellular milieu to coordinate pathogen clearance while avoiding damage to host cells and tissues. Glycans and glycan recognition drive and regulate immune responses at every level and provide inviting and often untapped opportunities for therapeutic development targeting immune dysregulation.

Among the most exciting recent findings is that humoral immunity is tuned by antibody glycosylation. Robert Anthony and Margaret Ackerman provided clinical and mechanistic insights related to IgE and IgG glycosylation. Allergen-specific IgE is absolutely required for allergic symptoms and disease. Unbiased examination of glycosylation patterns of total IgE from individuals with a peanut allergy and non-atopic individuals revealed altered glycosylation – an increase in sialic acid content – on IgE from allergic subjects (Shade et al., 2020). Selective sialic acid removal from IgE lessened effector-cell degranulation and anaphylaxis in allergic disease models. These findings make IgE glycosylation a promising target for therapeutic modulation.

Human IgG Fc glycans also correlate with disease outcomes, in both infectious and autoimmune diseases (Cobb, 2020). This appears to be due to the ability of various IgG Fc glycoforms to drive distinct Fc-dependent mechanisms and immune outcomes, from activating immunity to supporting tolerance. Intriguingly, glycoform expression may be specific for the antigen eliciting the response. Evidently, B-cell glycan biosynthetic enzymes respond to the antigen and regulate Fc glycosylation to tune the downstream response (Larsen et al., 2021). For both IgE and IgG, the technology has been developed to create designer immunoglobulin glycans, thereby modulating immune responses for therapeutic benefit.

Glycosylation of cell surface molecules on immune cells also regulates immune outcomes. Michael Demetriou described how the patterns of N-glycosylation on cell surface glycoproteins control the distribution, clustering, and surface residency of immune regulatory glycoproteins in a predictable manner. The mechanism involves glycan-binding proteins called galectins that, when N-glycans are sufficiently abundant and branched, form a cell surface lattice of immune regulatory molecules on both T cells and B cells (Mortales et al., 2020). Insufficient branching of N-glycans can result in autoimmune sensitivity, for example, in multiple sclerosis and autoimmune diabetes (Brandt et al., 2021). Remarkably, oral administration of the sugar N-acetylglucosamine in human subjects increases N-glycan branching, raising the hope that dietary supplementation may reduce autoimmunity.

Whereas GlcNAc-induced N-glycan branching regulates cell surface residency on immune cells, the same single sugar is dynamically attached to and removed from specific serine and threonine residues of cytoplasmic, nuclear, and mitochondrial proteins. This modification (O-GlcNAc) modulates protein and cell functions in immunity, cancer, neurodegeneration, and diabetes (among others) and is regulated by a single transferase (OGT) and glycosidase (OGA). Christina Woo shared new technologies to fuse nanobodies to these enzymes to modulate the O-GlcNAc residency of a particular protein or protein site (Ramirez et al., 2020; Ge et al., 2021). These methods promise to allow interrogation of the roles of O-GlcNAc on target proteins and to decode O-GlcNAc regulation.

Once an immune response is elicited, it must be controlled to avoid pathology due to the activated immune cells causing host tissue damage. Glycans play a role in this process as well. Ronald Schnaar described the 14-member family of human glycan-binding proteins (GBPs) called Siglecs, most of which are expressed on the surfaces of overlapping sets of immune cells and most of which dampen immune responses *via* intracellular immunoreceptor tyrosine-based inhibitory motifs (Duan and Paulson, 2020). When inhibitory Siglecs on activated immune cells encounter their native glycan ligands on target tissues, the immune cells apoptose or are otherwise inhibited, halting the ongoing immune event. Based on these findings, Siglecs are being targeted therapeutically as immune checkpoint inhibitors (Youngblood et al., 2020).

## Tools and Resources for Glycobiology

Despite significant advances in the study of glycosylation, there is much to be learned regarding the biological roles of these highly diverse molecules. For example, the human glycome is predicted to be vast: Some estimates suggest well over 7,500 unique structures, which require more than 700 genes for synthesis (Cummings, 2009). These structures are further diversified with additional modifications, including sulfation, methylation, and acetylation, which can directly impact or alter the function of individual glycans. Progress in the fields of glycomics and glycobiology has been limited by technical challenges in glycan sequencing and glycan synthesis, and insufficient tools to characterize the temporal and spatial expression of glycan determinants at high resolution. These barriers are coming down, providing enhanced opportunities to decode glycosylation function in physiology and pathology.

Determining the sequences of glycan structures remains a highly specialized technique that requires multiple orthogonal approaches, microgram amounts of material isolated from proteins or lipids, and does not capture the spatial and temporal nature of the glycan itself. Glycan synthesis also presents significant challenges. Functional synthetic glycans must retain the correct linkages between sugars in the correct stereochemical orientation. Clay Bennett introduced ways in which the stereochemical outcome of glycosylation can be controlled using methods that are accessible to novice synthetic chemists and scalable (Zhuo et al., 2019; Ling and Bennett, 2020). Democratizing glycan synthesis can advance glycomedicine, as evidenced by the development of synthetic glycans capable of targeting drug resistant pathogens and a potentially new class of antibiotic drugs.

To define the localization of glycans, identify their components, and explore their functions in biological tissues, the most commonly utilized tools in the glycobiologist’s toolkit are lectins and monoclonal antibodies (mAbs). Lectins, which are GBPs found in animals and plants, are used extensively, although their broad specificity can limit their utility. For example, three plant lectins are commonly used to distinguish between two biologically important structures: α2-3 linked sialic acid (bound by MAL, *Maackia amurensis* lectin I and II) and α2-6 linked sialic acid (bound by SNA, *Sambucus nigra* agglutinin). However, MAL-I and MAL-II also bind 3-O-sulfated determinants and SNA binding can be inhibited by lactose or galactose. Thus, interpretation of such experiments always requires caveats. Addressing this challenge, Lori Yang presented exciting technology in development to engineer more specific GBPs called Lectenz^®^. These proteins are engineered from carbohydrate-processing enzymes that exhibit high specificity and affinity for monosaccharides and glycosidic linkages. By eliminating catalytic activity and enhancing affinity using directed evolution informed by computational predictions of known molecular interactions, enhanced GBPs are generated. In theory, this innovative approach could convert any glycoactive enzyme to a binding reagent that is far more specific than traditional lectins, providing valuable reagents to further our understanding of glycobiology (Angel et al., 2021; Büll et al., 2021).

Monoclonal antibodies are another powerful tool to examine glycan localization and function. However, the mAbs available to researchers bind only a small fraction of the predicted glycan epitopes within the human glycome and fewer than a third of them are reliably available from commercial sources (according to a survey of the Database for Anti-Glycan Reagents); the situation is even worse for mAbs that specifically recognize microbial glycans. The paucity of such commercial reagents forces many laboratories to produce their own mAbs, an expensive solution that perpetuates problems of availability. Finally, due to the similarity of the human and mouse glycomes, human glycan structures are often not immunogenic and result in the production of IgM mAbs with broader specificity. To address these obstacles, Tanya McKitrick is developing “smart” anti-glycan reagents (SAGRs) by immunizing the sea lamprey, *Petromyzon marinus*, and then producing recombinant lamprey antibodies with a mouse/rabbit Fc for detection purposes (McKitrick et al., 2020, 2021). Lampreys have evolved an alternative adaptive immune system that occurs only in jawless vertebrates and uses a family of highly diverse, single-chain antibody-like proteins called variable lymphocyte receptors B (VLRBs). The potential diversity of SAGRs exceeds that of antibody production in mice, studies to date have identified over 25 VLRBs which can discriminate between glycosidic linkages, functional groups, and monosaccharides. These VLRB antibody sequences are publicly available in GenBank.

A further exciting area of tool development relates to protein-glycan interactions. Greg Hudalla discussed how galectins recognize glycans of the cell surface and extracellular matrix and thereby modulate biological processes, including those relevant to inflammation and infection. His group has developed peptide-based platforms to engineer multivalent scaffolds to influence galectin interactions at the cellular level (Restuccia et al., 2015; Farhadi et al., 2021). Beyond defining key biological interactions, these approaches have potential application in areas, including signaling, apoptosis, and drug delivery.

## Discussion

The workshop presentations briefly reviewed above highlight the importance of research in glycobiology for the advancement of fundamental knowledge and human health. Approaches from glycome profiling to glycan engineering have deepened our understanding of glycan mediated host-pathogen interactions and regulation of host immunity. This understanding in turn increases our ability to develop feasible approaches for diagnosis, treatment, and prevention of infectious disease as well as for control of both protective and dysregulated immune responses.

Further development of tools and resources to help characterize, localize, and engineer glycans and glycan-binding proteins will accelerate discovery and application in both infection and immunity. Studies of infection will benefit from analysis and synthesis of microbial glycans, examination of the host activities and glycoconjugates that modulate events at the host-pathogen interface, the use of microbe diversity to uncover new processes and cellular interactions, and the expansion and availability of glycan arrays that reflect the diversity of microbes and their host niches. Studies of immunity will benefit from the ability to analyze, create, and regulate specifically glycosylated antibodies to control immune outcomes; therapeutically regulate cell surface glycans to modulate their responsiveness target intercellular glycosylation to modulate signaling pathways; and target native immune inhibitory pathways with glycans. Robust support of these efforts will continue to yield exciting scientific discoveries and improved human health.

## Author Contributions

RS and TM drafted individual sections and edited the manuscript. TD drafted the remainder of the manuscript and edited the manuscript. All other authors edited the manuscript.

## Funding

Glycobiology studies in the Doering group are funded by the National Institute of Allergy and Infectious Diseases (NIAID) R01 AI135012 and in the Schnaar group by NIAID U19 AI135443. The May 27–28, 2020, NIH workshop on Glycoscience and Immunology at the Crossroads of Biology was funded by NIAID and the National Institute of Dental and Craniofacial Research (NIDCR), with meeting support from NIAID.

## Conflict of Interest

MD is an inventor on a patent for use of GlcNAc in MS and co-founded Glixis Therapeutics, a company that was developing analogs of GlcNAc for MS and other autoimmune diseases. GAH is a founder and stockholder of Anchor Biologics, Inc. and is an inventor on patents filed by and awarded to the University of Florida. Harvard University has filed a patent application on the nanobodies mentioned in conjunction with the work of CMW, who is an inventor of the patent. LY was employed by the company Lectenz Bio, which has joint patents with the University of Georgia Research Foundation, Inc. related to the research discussed. Lectenz Bio has licensed the patents, and LY is a named inventor.

The remaining authors declare that the research was conducted in the absence of any commercial or financial relationships that could be construed as a potential conflict of interest.

## Publisher’s Note

All claims expressed in this article are solely those of the authors and do not necessarily represent those of their affiliated organizations, or those of the publisher, the editors and the reviewers. Any product that may be evaluated in this article, or claim that may be made by its manufacturer, is not guaranteed or endorsed by the publisher.
